# Myofascial trigger points and innervation zone locations in upper trapezius muscles

**DOI:** 10.1186/1471-2474-14-179

**Published:** 2013-06-08

**Authors:** Marco Barbero, Corrado Cescon, Andrea Tettamanti, Vittorio Leggero, Fiona Macmillan, Fiona Coutts, Roberto Gatti

**Affiliations:** 1 Department of Health Sciences, University of Applied Sciences and Arts of Southern Switzerland, SUPSI, Manno, Switzerland; 2 Queen Margaret University, School of Health Sciences, Edinburgh, United Kingdom; 3 Vita-Salute San Raffaele University, School of Physiotherapy, Milan, Italy

**Keywords:** Myofascial trigger point, Myofascial pain, Innervation zone, Endplate, Surface EMG

## Abstract

**Background:**

Myofascial trigger points (MTrPs) are hyperirritable spots located in taut bands of muscle fibres. Electrophysiological studies indicate that abnormal electrical activity is detectable near MTrPs. This phenomenon has been described as endplate noise and it has been purported to be associated MTrP pathophysiology. Thus, it is suggested that MTrPs will be overlap the innervation zone (IZ). The purpose of this work was to describe the location of MTrPs and the IZ in the right upper trapezius.

**Methods:**

We screened 71 individuals and eventually enrolled 24 subjects with neck pain and active MTrPs and 24 neck pain-free subjects with latent MTrPs. Surface electromyography (sEMG) signals were detected using an electrode matrix during isometric contraction of the upper trapezius. A physiotherapist subsequently examined the subject’s trapezius to confirm the presence of MTrPs and establish their location. IZ locations were identified by visual analysis of sEMG signals. IZ and MTrPs locations were described using an anatomical coordinate system (ACS), with the skin area covered by the matrix divided into four quadrants.

**Results:**

No significant difference was observed between active and latent MTrPs locations (P = 0.6). Forty-five MTrPs were in the third quadrant of the ACS, and 3 were included in second quadrant. IZs were located approximately midway between the seventh cervical vertebrae and the acromial angle in a limited area in the second and third quadrants. The mean distance between MTrP and IZ was 10.4 ± 5.8 mm.

**Conclusions:**

According to the acquired results, we conclude that IZ and MTrPs are located in well-defined areas in upper trapezius muscle. Moreover, MTrPs in upper trapezius are proximally located to the IZ but not overlapped.

## Background

Musculoskeletal impairments are the leading cause of work disability in middle-aged adults, and low back pain is the most common form [1]. There are two broad clinical categories of musculoskeletal impairments: articular and non-articular [2]. The articular group includes joint diseases (intra-articular) involving inflammation or injuries due to trauma or degenerative processes. Typical examples are rheumatoid arthritis or shoulder instability. The non-articular group includes disorders that primarily affect soft tissue (peri-articular), such as muscles, ligaments, and tendons. Examples are fibromyalgia, myofascial pain syndrome, and tendonitis. It is thought that up to 85% of patients that visit pain clinics complain of non-articular musculoskeletal impairments such as the myofascial pain syndrome [3]. The first authors who systematically described the myofascial pain syndrome were Travell and Simons, who theorised that this painful condition is due to the presence of myofascial trigger points (MTrPs) [4].

MTrPs are hyperirritable points located within a taut band (TB) of skeletal muscle that cause referred pain, local tenderness, and sometimes autonomic changes [4,5]. An active MTrP is characherized by spontaneus pain or pain response to movement, while a latent MTrP is a sensitive spot with pain only elicited in response to compression [5]. The MTrP diagnosis requires detailed history taking and physical examination to confirm the presence or absence of an original set of diagnostic criteria (i.e., taut band, spot tenderness, referred pain, pain recognition, local twitch response) [5].

MTrP pathophysiology appears to be associated with the motor endplate zone [6-10]. This region, also known as the innervation zone (IZ), is where the α-motor neuron divides into a number of branches and synapses onto target muscle fibres. The IZ is usually described as being in the middle region of the muscle belly [11], but more complex IZ spatial distibutions have been reported in muscles with unipennate or multipennate fibre arrangements (e.g., gastrocnemius and soleus) [12-14]. Needle electromyography (EMG) studies have demonstrated that MTrPs contain minute loci that produce characteristic low-amplitude electrical activity [10,15,16] (i.e. active locus), which is described by in the literature as spontaneous electrical activity (SEA).

The origin of SEA has been extensively debated among experts. Initially, Hubbard and Berkoff attributed the source of SEA action potentials to intrafusal muscle spindles located near MTrPs [15]. Later, Simons considered previous work by Liley [17] and hypothesised that SEA originates from motor endplates and defined it as endplate noise [8].

In support of the last hypothesis, a needle EMG study showed that endplate noise was more prevalent in MTrPs than in adjacent sites [10].

This “motor endplate” hypothesis was also tested by Kuan et al., who injected MTrPs with botulinum toxin type A to block acetylcholine release into the synaptic cleft and found that the injection diminished SEA in MTrPs [18].

Finally, the described electrophysiological findings have been correlated with histological changes [19,20] and local biochemical alterations [21,22] (e.g., inflammatory mediators, neuropeptides, catecholamines, and cytokines) to support MTrP pathophysiology.

The described experimental findings of MTrPs suggest that they are located close to the IZ [2,16]. In a research context aimed to clarify the MTrP etiology a confirmation of the overlapping between MTrP and IZ will help to clarified their interaction (i.e. the active locus and the sensitive locus) [23]. Furthermore manual identification of MTrPs could become useful to optimize treatments addressed to the IZ, like botulinum injections for both cervical dystonia and myofascial pain [24,25].

A technology useful for detecting the IZ in vivo has recently proposed [26-29], and to-date, no study has assessed both MTrP and IZ locations. Therefore, the purpose of this work was to describe MTrP and IZ locations in the upper trapezius muscle.

## Methods

All experimental sessions were conducted at the laboratory of movement analysis of the San Raffaele Scientific Institute in Milan, Italy. The Internal Ethical Committee approved the protocol. All participants signed an informed consent form before enrolling in the study.

### Participants

Twenty-nine patients with chronic mechanical neck pain and 42 pain-free subjects with negative histories for neck/shoulder pain were screened at the laboratory of movement analysis at the San Raffaele Scientific Institute in Milan. Patients were recruited through the Rehabilitation Service of San Raffaele Hospital, and pain-free subjects consisted of San Raffaele Scientific Institute employees. Mechanical neck pain was defined as pain elicited by active cervical spine movements and perceived anywhere in the posterior region of the cervical spine, from the superior nuchal line to the first thoracic spinous process [30]. Neck pain subjects were screened as they usually show MTrPs in upper and mid trapezius muscles [4].

Patients had to meet the following inclusion criteria: mechanical neck pain, neck pain history lasting more than 3 months, an active or latent MTrP in the right upper trapezius. Exclusion criteria were: positive history for neurological or rheumatic disorders, radiculopathy, fibromyalgia, joint disorders, whiplash in the previous 6 months, pregnancy, clinical depression and body mass index ≥30. Concomitant painful disorders and psycho emotional distress were ruled out to avoid adverse effects and confounding factors during the MTrP palpation procedure. Overweight subjects were excluded because an excessive subcutaneous fat layer thickness can limit the MTrP palpation.

Forty-eight subjects were enrolled and analysed: 18 patients with chronic neck pain and active MTrPs, 6 patients with chronic neck pain and latent MTrPs, and 24 pain-free subjects with latent MTrPs. The following subjects were excluded from the study: 16 didn’t show any MTrP in the right upper trapezius muscle, 3 were excluded due to a positive history for neurological disorders, and 4 showed an MTrP located outside the area covered by the electrode matrix.

MTrPs were detected by a physiotherapist with 10 years of clinical experience specialising in myofascial pain syndrome diagnosis and management.

The physiotherapist explored the upper trapezius area using a flat palpation technique [4]. Only neck pain patients with an active or latent MTrP and pain-free subjects with latent MTrP were considered for further analysis. Diagnostic criteria for active MTrP were: the presence of a TB within the upper trapezius muscle and at least one of the following clinical signs; the presence of a spot tenderness (SP) within the TB, the reproduction of pain complains during mechanical stimulation of the SP, and the reproduction of a referred pain with mechanical stimulation of the SP [31]. The presence of a local twitch response during snapping palpation of the TB was considered confirmatory criteria [5]. Diagnostic criteria for latent MTrP were TB and SP.

### Equipment

Surface electromyographic signals (sEMG) were detected using a matrix (model ELSCH064) composed by 4 columns of 13 electrodes and 1 column of 12, with 8 mm interelectrode distance (IED) designed by LISiN at Politecnico di Torino and manufactured by OT Bioelettronica, Torino, Italy. The matrix was fixed on the skin with double adhesive tape. The cavities corresponding with the electrode were filled with 20 μl conductive paste using a spatula to ensure proper electrode–skin contact. sEMG signals were amplified with an EMG-USB amplifier (LISiN - OT Bioelettronica, Torino, Italy - bandwidth 10–750 Hz; adjustable gain between 500 and 10,000; sampling frequency 2048 Hz; 16 bits A/D converter). Samples were visualised during acquisition and stored on a personal computer OT-Biolab software (OT Bioelettronica, Torino, Italy).

**Figure 1:**
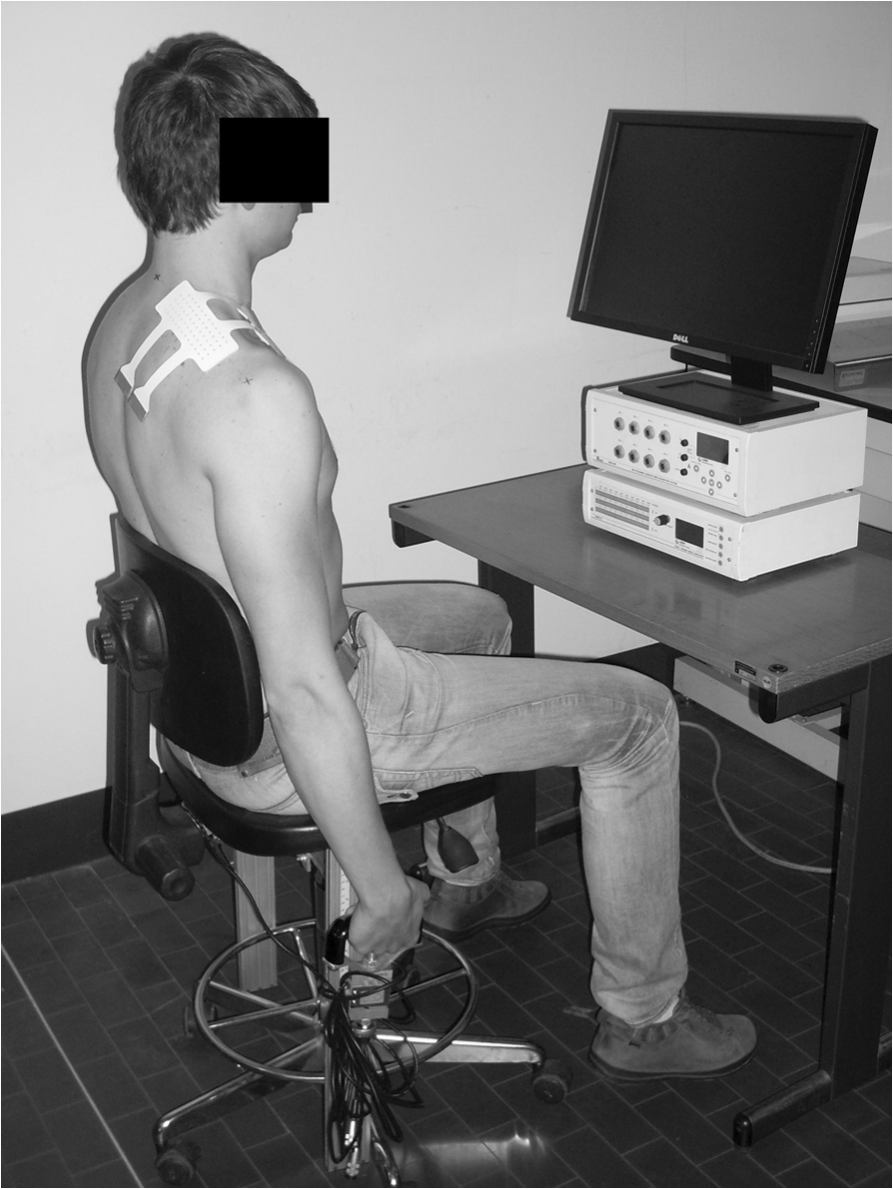
**Experimental setup.** Subjects sat in a custom made chair with a load cell connected to the right handle and were connected to an electrode matrix (model ELSCH064, designed by LISiN at Politecnico di Torino and manufactured by OT Bioelectronica, Torino, Italy) placed on the upper trapezius. They viewed the visual feedback device (MISO II, LISiN, OT Bioelettronica, Torino, Italy) during the experiment.

In order to measure the torque exerted by the upper trapezius muscle, subjects were asked to sit on a custom designed chair and hold both chair handles (Figure 1). The handle on the right side was fixed to a load cell in order to measure the force exerted during shoulder elevation. Force signals were acquired and amplified (bandwidth 0–80 Hz) using an MISOII amplifier (LISiN - OT Bioelettronica, Torino, Italy). Subject feedback was provided by a bar of light-emitting diodes indicating the percentage of the maximum voluntary contraction (MVC) reached during each shoulder elevation. MTrP pain pressure threshold (PPT) was assessed with a pressure algometer (Wagner Instruments, Greenwich, CT, USA) at an application rate of approximately 1 kg/s for each MTrP. The algometer had a rubber tip with a 1-cm^2^ contact area.

### Procedure

The day after enrolment and immediately prior to the experimental procedure, patients with neck pain completed a Neck Disability Index (NDI) and a visual analogue scale (VAS). The mean pain experienced in the last week was reported. Pain-free subjects started the experimental procedures immediately.

Prior to each experimental session, the same operator used a surgical pen to draw a standardised anatomical coordinate system (ACS) on the right shoulder of subjects while they were seated on the data acquisition chair. The ACS consisted of a line between the spinal process of the seventh vertebrae and the acromial angle (X-axis), and a second line perpendicular to the first one and passing through its midpoint (Y-axis). The centre of the electrode matrix was placed on the intersection between the axes with the matrix columns parallel to the X-axis line so that the skin area covered by the matrix was divided into four quadrants (first, second, third and fourth).

To measure MVC, subjects were instructed to perform a shoulder elevation task by pulling the chair’s handles upwards. The MVC force level was determined as the maximum of three isometric contractions. Two minutes of rest were provided between the maximum exertions.

Each subject was given instructions and allowed to practice for 5 min in order to learn to keep the force level at 20% MVC using the feedback provided. Following this, sEMG signals were acquired for 20 seconds at 20% MVC isometric contraction. After the electrode matrix was removed, the expert physiotherapist performed an examination of the upper trapezius muscle using flat palpation techniques in order to locate the MTrP according to the established diagnostic criteria [31], and their location was marked on the skin using a custom designed stamp (a 1-cm^2^ circle with a dot in the centre). The dot in the centre was overlapped with the SP on the TB, and its distance from the X- and Y-axes of the ACS was measured with a ruler.

**Figure 2:**
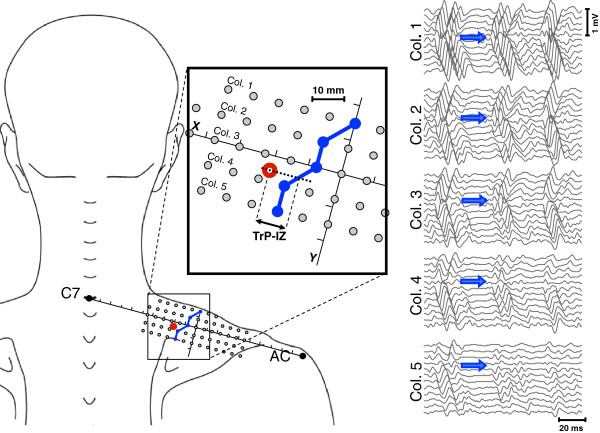
**Electrode matrix placement.** The red dot indicates an MTrP in the upper trapezius and the blue line shows the IZ location over the area covered by the electrode matrix. The IZ was typically located medially to the Y-axis. TrP-IZ is the distance between SP in the MTrP region and the IZ line. For each of the five columns of the matrix EMG signals are reported and IZ locations indicated using a blue arrow. The IZ location was detected where EMG signals showed minimal amplitude and/or phase reversal. AC, acromial angle; C7, spinous process of the seventh vertebrae; SP, spot tenderness.

An operator blinded to the MTrP location performed IZ visual analysis. The IZ was identified for each of the five columns of the bi-dimensional electrode matrix by means of visual analysis of the single differential sEMG signals [32]. The criteria to detect their location on each column were minimal amplitude and/or phase reversal of signals [27,28]. Subsequently, IZ locations were described according to ACS. To complete the IZ location over the upper trapezius muscle IZs detected on matrix columns were linked (linear interpolation) (Figure 2).

A PPT over the stamp was assessed for each MTrP. The PPT was defined as the minimum pressure that evokes local pain, and together with NDI (scale range, 0 to 50) and VAS (scale range, 0 to 100) provided detailed descriptions of investigated population.

### Statistical analysis

Data were tested for Gaussian distribution using Shapiro-Wilk test. The mean and SD of NDI, VAS and MTrPs PPTs were calculated in order to describe subject clinical parameters. IZ and MTrP locations were described in accordance to the four quadrants defined by the ACS. The distance between the SP and the IZ (TrP-IZ) was computed by tracing parallel to the X-axis, reflecting the upper trapezius fibre direction [33] and described as mean and SD. The TrP-IZ was measured along the X-axis because the intention was to intercept the IZs located in the same fibers that included the MTrP (Figure 2). The correlations among variables (NDI, PPT, VAS, X, Y, TrP-IZ) were examined using Pearson correlation coefficients. The Student’s t-test (α = 0.05) was used to compare the values of X, Y and TrP-IZ in active and latent MTrP, and finally to verify that TrP-IZ was significantly different form zero. SPSS version 19 (SPSS Inc., IBM Company, New York, NY, USA) was used to perform statistical analysis.

## Results

All the considered variables showed a normal distribution (VAS: P=0.280, NDI: P=0.70, PPT: P=0.154, X: P=0.595, Y: P=0.106, TrP-IZ: P=0.103). The enrolled neck pain patients showed a mean VAS score of 37.3 ± 15.2 and a mean NDI score of 11 ± 5 out 50. The mean PPT of the MTrPs was 2.6 ± 0.9 kg/cm^2^ (Table 1).

**Table 1:** Summarised results

**Subject**	**Group**	**MTrPs**	**VAS score**	**NDI score**	**PPT kg/cm**^**2**^	**X (mm)**	**Y (mm)**	**TrP-IZ (mm)**
1	H	L			2,9	−1	−1	10
2	NP	A	51	21	1,3	−1	−1,5	2
3	NP	L	21	8	2,1	−0,6	−1,5	10.5
4	NP	A	30	7	3,9	−2,4	−1,6	4
5	H	L			5,2	−1,2	0	0
6	NP	A	49	18	2	−1,8	−1,3	14
7	H	L			2,5	−1,9	−0,8	3
8	H	L			3,7	−2	−0,6	16
9	H	L			1,9	−1,7	−0,3	14.5
10	NP	A	19	5	1,6	−2,5	−0,7	17.5
11	NP	A	41	8	1,5	−2,3	−1,2	11
12	NP	A	49	18	2,9	−1,3	−1,3	1
13	NP	A	39	15	1,8	−1,6	−0,6	9
14	NP	A	30	8	1	−2,7	−1,4	10
15	NP	A	55	6	1,7	−1,4	−1,4	12
16	NP	A	21	10	2,4	−2,2	−0,3	18
17	H	L			2,4	−2,1	−0,5	19.5
18*	NP	A	-	-	-	−0,9	−2,1	-
19	H	L			3,1	−0,1	−1,5	6.5
20	NP	L	75	8	2,3	−1,8	−1,3	6
21	H	L			2,9	−2,9	−1,4	9
22	H	L			2,5	−1,6	−0,3	9
23	H	L			2,8	−3,1	0	7
24	NP	L	46	7	3,4	−1,7	−0,1	16.5
25	H	L			2,7	−1,5	0	11
26	H	L			1,8	−2,7	−0,6	16
27	H	L			1,6	−1,1	−0,6	3
28	H	L			2,7	−2	−0,4	14
29	H	L			4,8	−2,7	−0,7	19
30	H	L			2,6	−2,9	−1	20
31	H	L			2,5	−0,7	−0,5	0.5
32	NP	A	27	11	2,7	−2,2	0	10
33*	H	L	-	-	-	−1,4	−1,7	-
34	H	L			3,2	−1,4	−0,6	10
35	H	L			2,3	−1,4	−0,7	2.5
36	H	L			1,9	−2,7	−0,5	12.5
37	H	L			3,6	−2,1	0	13
38	H	L			2,1	−2	0,1	8
39	NP	A	20	4	3,2	−2,9	−0,7	17
40	H	L			2,3	−2,6	0	14
41	H	L			3,4	−1,5	−1,4	12
42	NP	A	67	16	2	−2,3	0,5	11
43	NP	A	39	16	1,7	−2,2	−0,6	15
44	NP	L	47	13	2,7	−1,4	−1,5	10.5
45	NP	A	34	9	2,2	−1,9	−1,5	8
46	NP	A	23	4	3,5	−0,5	0	1
47	NP	L	33	13	4	−1,5	−0,4	1
48	NP	A	34	11	3,8	−2,1	−0,6	16
49	NP	L	25	9	2,1	−2,7	0,5	20
50	NP	A	21	14	3	−1	−0,9	6
51*	NP	A	-	-	-	−2	−1,9	-
52*	NP	A	-	-	-	−1,8	−1,9	-
Mean±SD			37.3 ± 15.2	11 ± 5	2.6 ± 0.9			10.4 ± 5.8

Column two indicates patient group, *H* healthy group, *NP* neck pain group. Column three indicates MTrP status: *A* active MTrP, *L* latent MTrP. *Subjects who showed an MTrP located outside the matrix were excluded from the analysis.

No significant correlations were found among variables except between X and TrP-IZ (P<0,01).

**Figure 3:**
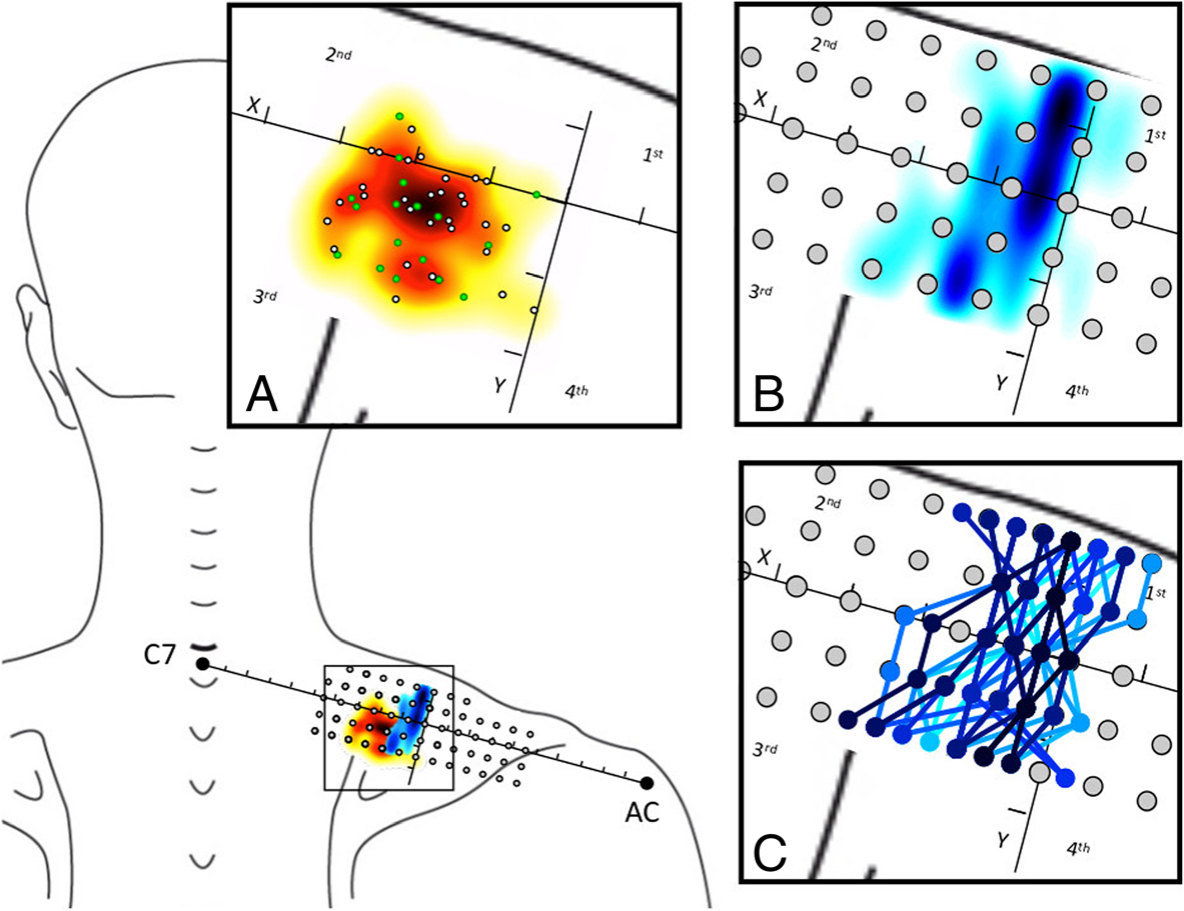
**Graphical representation of MTrPs and IZ location in the upper trapezius according to the ACS in 48 subjects. A**) Active MTrPs are represented as green circles and latent as white circles, and the colours indicate MTrP spatial densities (dark red spot indicates high trigger point density). **B**) IZ distribution, colour represents IZ density (dark blue area indicates high IZ density). **C**) Each IZ loction is represented by different colours link IZs detected on the matrix columns. AC, acromial angle; C7, spinous process of the seventh cervical vertebrae.

According to the ACS, 45 subjects showed an MTrP medially located with respect to the Y-axis, and all the MTrPs were located in the third quadrant except 3 that were located in second quadrant (Figure 3). No statistically significant difference was found for X (P = 0.6) or Y (P = 0.1) values between active and latent MTrPs.

The IZ was successfully detected in all subjects for each of the matrix columns and was not larger than 8 mm (i.e., the IED) (Figure 2). Typically, the IZ was located medially with respect to the Y-axis and not further than 2.4 cm from the Y-axis (i.e., 3 IED) and in an area that extended from second to the third quadrant. It was occasionally partially included in the first and fourth quadrants (7 out of 48 subjects) (Figure 3).

The mean TrP-IZ was 10.4 ± 5.8 mm, with no statistically significant difference between active and latent MTrPs (P = 0.6). TrP-IZ was significantly different from zero (P<0.01). Figure 3 shows both MTrPs and IZ locations according to the ACS.

## Discussion

The purpose of this work was to describe the location of MTrPs and IZs in the upper trapezius muscle. It is unclear whether the locations of the MTrPs and the IZs are closely related [2,16,23]. A study that investigates both these locations in the same subjects has not previously been undertaken. Previous studies have shown that the MTrP region includes active loci where it is possible to identify low-amplitude electrical activity (i.e., SEA), which was attributed to motor endplate dysfunction termed endplate noise. Additionally, high correlation between MTrP site irritability and endplate noise prevalence has been demonstrated by inserting an EMG needle [34]. The endplate noise searching procedure included the insertion of an intramuscular needle at the MTrP region identified during palpation [10]. As stated by the authors, this suggests an immediate proximity between the MTrP and the IZ [8,10,16,18].

Results indicated that MTrPs were located in a well-defined area of the upper trapezius with a PPT that was clearly lower than in normal subjects [35] and showing values similar to those reported in previous studies [10,34,36].

In the current study, the investigated area was the inferior part of upper trapezius and a portion of the upper part of the mid trapezius where fibres were described to be horizontally oriented [33]. This anatomical region is where patients with neck pain typically report tenderness and where both active and latent MTrPs are frequently observed [37]. The data describe an area for MTrP location in upper trapezius that is similar to the MTrP chart proposed by Simons and Travell, which also matches the results of a recent study that used the same ACS [38]. MTrPs in the upper trapezius appear to have a stereotyped location, and clinicians could use our ACS to guide their palpatory examinations.

No significant correlations were found among the considered variables except between X and TrP-IZ, that obvious considering how TrP-IZ was computed. Additionally, no significant differences were observed for either X or Y values between active and latent MTrPs. A similar location for active and latent MTrP would support, as described by Simons, a natural course for myofascial pain that includes a subclinical stage in which the MTrPs are not spontaneously painful (i.e., latent) [5]. We screened 42 neck pain-free subjects with a negative history for arm/shoulder complains, and just 16 were negative for TBs with SPs. The common presence of TBs in pain-free subjects [37,39,40] and the similar location for active and latent MTrPs seem to suggest that TBs could be considered as a necessary precursor to the development of MTrP. It is likely that stress factors (e.g., muscle overload or emotional distress) could be involved in progression from latent to active [5]. This was recently confirmed by Shah et al., who demonstrated that active and latent MTrPs contain the same biochemical substances [22] (bradykinin, substance P and serotonin), and that their concentration is lower in latent MTrPs compared with active MTrPs. In the current study, SP compression failed to evoke complaints (i.e., a negative PR criteria) in just 6 of 24 subjects with neck pain, suggesting that MTrPs in the upper trapezius frequently contribute to neck pain. The presence of MTrPs should not be overlooked when examining subjects with painful conditions; moreover, high MTrP prevalence has been reported in several selected patient populations [41-44].

The electrode matrix covered a 30.72 cm^2^ area (9.6 cm × 3.2 cm), and the IZ can be approximately drawn on the skin as a straight line that runs orthogonally to the upper trapezius fibres and tends to curve medially towards the spine in its caudal part. A similar distribution for the IZ in upper trapezius was previously reported by Saito el al. in three healthy subjects [29]. However, this is the first time that IZ location was investigated in a large group of subjects considering the area that extends over the muscle surface. The same experimental setting was applied a in previous study that did not focused on the IZ morphology [26]. It should be noted that there was limited variability for the IZ location in the upper trapezius, and our results support the generally accepted principle that muscles with parallel fibres contain IZs in the midbelly [11].

Our findings confirm that the MTrPs in the upper trapezius are located in proximity of the IZ but do not overlap; rather, they are about 10 mm apart. In contrast with previous investigations, we observed distinct locations for IZs and MTrPs, but it is important to note that we investigated a different region of the upper trapezius, from previous studies. Interestingly, MTrPs were not equally distributed along the IZ and only affected specific groups fibres in the upper trapezius muscle.

The described close spatial relationship between IZ and MTrPs can be potentially useful to guide treatments targeting the IZ. As for example botulinum toxin injection in various pain conditions including muscle spasticity, cervical dystonia, headache and myofascial pain [24,45].

A few limitations need to be taken into account when interpreting the results of this study.

We identified MTrP locations using SPs located on TBs using palpation, similar to previous studies [10,34]. Although this method has been demonstrated to reliably locate MTrPs in the upper trapezius, we are aware that this only provides an approximation ranging from a few millimetres to 1.5 centimetres [36]. Also our detection methodology for the IZs and MTrPs gives bi-dimensional locations on the skin, rather than a 3-dimensional location. Finally, it is important to note that the electrode array with 8-mm IED provides an approximation of about 4 mm when locating the IZ [26].

## Conclusion

MTrP and IZ locations were described according to the ACS in all enrolled subjects. MTrPs were located in well-defined areas of the upper trapezius, showing a typical location with a mean TrP-IZ of 10 mm. MTrPs in upper trapezius are proximally located to the IZ but not overlapped. These results provide an interesting insight for future research regarding the mechanism underlying the MTrP iperalgesia. Moreover, the anatomical reference system proposed in this study may help clinicians identify these areas.

## Abbreviations

ACS: Anatomical coordinate system; EMG: Electromyography; IED: Interelectrode distance; IZ: Innervation zone; MTrP: Myofascial trigger point; NDI: Neck disability index; PPT: Pain pressure threshold; SEA: Spontaneous electrical activity; SP: Spot tenderness; sEMG: Surface electromyography; TB: Taut band; TrP-IZ: Distance between the spot tenderness and the innervation zone; VAS: Visual analogue scale.

## Competing interest

I certify that neither I nor any co-authors have actual or potential conflict of interest in relation to this article exists.

## Authors' contributions

MAB conceived the study, partecipated in its design, carried out the data collection, carried out the stastistical analysis, and drafted the manuscript. CE performed the data processing and helped to draft the manuscript. TEA,VIL helped in planning the experimental sessions and supported the data collection. FIM, FIC partecipated in the design of the study and helped to draft the manuscript. ROG: helped in conceiving the study, coordinated the experimental sessions, and helped to draft the manuscript. All authors read and approved the final manuscript.

## Pre-publication history

The pre-publication history for this paper can be accessed here:

http://www.biomedcentral.com/1471-2474/14/179/prepub
